# Design of 4 × 4 Low-Profile Antenna Array for CubeSat Applications

**DOI:** 10.3390/mi14010180

**Published:** 2023-01-10

**Authors:** Diana Alondra Jiménez, Alberto Reyna, Luz Idalia Balderas, Marco Antonio Panduro

**Affiliations:** 1 Electrical and Electronic Engineering Department, University Autonomous of Tamaulipas, UAMRR, Reynosa 88779, Mexico; 2 Electronics and Telecommunications Department, CICESE Research Center, Ensenada 22860, Mexico

**Keywords:** antenna array, CubeSat, fifth generation (5G), high gain, miniaturization, nanosatellite

## Abstract

This paper presents a low-profile microstrip antenna with high gain for fifth-generation (5G) CubeSat applications. The proposed design consists of 16 miniaturized patch antennas distributed in a uniform 4 × 4 topology with a feeding network on Rogers TMM10 substrate. The antenna array was simulated in CST Studio Suite^®^ software and fabricated for performance testing on the CubeSat structure. The prototype works perfectly from 3.46 GHz to 3.54 GHz. The simulated and measurement results reveal remarkable performance. The design obtained a measured gain of 8.03 dBi and a reflection coefficient of −17.4 dB at the center frequency of 3.5 GHz. Due to its reduced dimensions of 10 × 10 cm, this design is an excellent alternative for mounting on a CubeSat structure as it combines efficient performance with a low profile.

## 1. Introduction

Given the demand for increasingly efficient global communication with great coverage and a long range, the development of low earth orbit (LEO), medium earth orbit (MEO), geostationary earth orbit (GEO), and high-altitude platform station (HAPS) satellite systems is required [1,2]. In the last two decades, the development of these telecommunications satellite systems has experienced a great boom, since it has evolved beyond the use of large satellites to include the design, manufacture, and launch of new projects of smaller size and cost. In this sense, nanosatellites, whose mass is between 1 and 10 kg, stand out [3]. A CubeSat is a standardized form of this type of satellite. In 1999 its standard [4] was created, thus initiating a new era in satellite design. Currently, revision number 14 is available, where a standard CubeSat unit or “1U” is defined as a cube-shaped structure with a restricted volume of 10 cm × 10 cm × 10 cm and a mass of up to 1.33 kg [5].

A key component of the CubeSat communication system is the antenna. This device is used to send data from the nanosatellite to the ground station and to receive commands from it. However, its design is very challenging, as it must meet the size and mass restrictions of the CubeSat standard while offering high gain.

On the other hand, frequency is also a key parameter to be considered. In this case, with the publication of new specifications for 5G mobile technologies, and with the conclusion of the first complete set of standards, a new area of technological development opportunity has been opened for nanosatellites that operate in their frequency bands. Since Release 15 of 3GPP (3rd Generation Partnership Project), the radio frequency range from 3.3 GHz to 4.2 GHz was enabled as a new band for 5G [6]. This frequency band is suitable for providing narrowband IoT services with low-orbit nanosatellites as mentioned in Release 17 for Narrow Band IoT/extended Machine Type Communication standards [7]. In this sense, the modern antenna design trends for nanosatellites consider the aspects of low-profile, high-gain, and 5G frequency ranges as mentioned in [8].

Several studies have been presented in this research area with different perspectives, mainly based on four types of antennas: monopole [9,10,11], dipole [12], helical [13], and microstrip patch. The application of patch antennas for 5G is in full development for CubeSats [14,15,16], mobile communications [17,18,19,20], and the use of new frequency bands for this technology [21,22,23]. This type of antenna stands out for its practicality for application in nanosatellites through the design of antenna elements or arrays focused on the operating frequencies of the ultra-high frequency (UHF,) L, S, X, Ku, and K bands. In [24], the design and analysis of microstrip antenna arrays for meteorological nanosatellites for UHF uplink were demonstrated. Later, UHF and S-band antenna arrays for data retransmission were presented in [25]. The work in [26] presented the design and compatibility analysis of a solar panel-integrated UHF antenna. On the other hand, the research in [27] focused on the design and characterization of an antenna that operates in the S-band (2.4 GHz). This antenna sends all the ADS-B (Automatic Dependent Surveillance-Broadcast) data collected to the ground to automatically and periodically transmit air traffic information for each aircraft, including identity number, position, speed, and destination during all phases of flight to avoid collisions. Moreover, this antenna showed return loss values of −18.5 dB, a bandwidth of 163 MHz, and a gain of 6.08 dB. Concerning the X band, a design for a compact antenna for isoflux radiation with circular polarization operating in the X band (8–8.4 GHz) was presented in [28]. Moreover, an array of 25 elements operating in the central frequency of 8.2 GHz with a realized gain of −10 dB was proposed in [29]. In [30], the design of a 4 × 4 element microstrip-phased antenna array for a satellite application of the Internet of Things (IoT) was developed, operating on the center frequency of 8.21 GHz. Additionally, for the K band, the design of a patch antenna operating at 22.5–23.5 GHz with high gain for low Earth orbit interconnection between nanosatellites, forming an array of 4 × 4 elements with a gain of 21.8 dBi, was presented in [31].

The main contribution of this work is the design of an antenna array based on microstrip technology with a low profile for high-gain wireless links at low frequencies of 5G in CubeSat communications. To this end, the design presents the use of miniaturized antenna elements that allow for its implementation in the limited structure of a nanosatellite.

## 2. Antenna Design Methodology

### 2.1. Antenna Element Design

The initial antenna element on which the design is based consists of a microstrip patch operating at 3.5 GHz on a Rogers TMM10 substrate with a permittivity of 9.2. This antenna has a compact dimension of 22 mm × 27 mm × 1.270 mm. Its parameters were calculated based on the transmission line method presented in [32], where the effective dielectric constant is calculated by:

(1) $$\varepsilon_{\mathrm{reff}}=\frac{\varepsilon_{r}+1}{2}+\frac{\varepsilon_{r}-1}{2}{\left(1+12\frac{h}{W}\right)}^{-1/2},$$

where *ε_r_* is the dielectric constant of the substrate, *h* is the height of the substrate, and *W* is the width of the patch. The length of the patch is obtained by:

(2) $$\Delta L=0.412h\frac{\left(\varepsilon_{\mathrm{reff}}+0.3\right)\left(W/h+0.264\right)}{\left(\varepsilon_{\mathrm{reff}}-0.258\right)\left(W/h+0.8\right)},$$

(3) $$L=\frac{c}{2\;fr\;\sqrt{\varepsilon_{\mathrm{reff}}}}-2\Delta L,$$

where “∆*L*” is the length of the transmission line, *L* is the length of the patch, c is the speed of light in free space, and *fr* is the resonant frequency of the antenna. The width of the patch can be represented by:

(4) $$W=\frac{c}{2fr}\sqrt{\frac{2}{\varepsilon_{r}+1}}.\;$$

The ground plane dimensions can be calculated with the help of the following equations:

(5) $$L_{g}=6h+L$$

(6) $$W_{g}=6h+W$$

**Figure 1:**
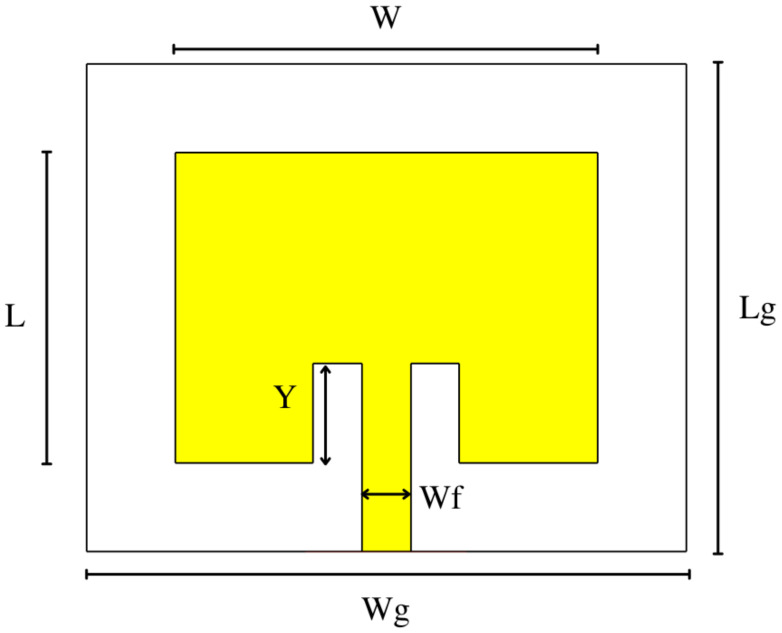
Initial antenna element design.

The structure was modeled in the EM 3D CST Studio Suite^®^ analysis software (Version 2019, Dassault Systèmes, Vélizy-Villacoublay, France) as shown in Figure 1. Its physical dimensions were determined to be the following values: *L_g_* = 22 mm, *W_g_* = 27 mm, *L* = 14 mm, *W* = 19 mm, *W_f_* = 2.2 mm, and *Y* = 4.5 mm.

### 2.2. Antenna Element Miniaturization

Multiple antenna elements are necessary to achieve the requirement of the high gain wireless transmission of CubeSats. Nonetheless, the nanosatellite structure is very tiny. Therefore, it is mandatory to reduce the size of the designed element to satisfy this need. The slot miniaturization technique was applied to the patch antenna. The fundament of this technique consists of slotting the patch. In this way, it is possible to have a longer perimeter of the patch, which allows it to resonate at a low frequency without increasing the element size. That is, by adding more slots, the perimeter of the patch increases, but the structure is reduced. Then, the optimization of the antenna dimensions with the CST Studio Suite^®^ software was run. The details of the optimization strategy were as follows: the Particle Swarm Optimization algorithm was used with a swarm size of 30, a maximum number of iterations of 15, and a maximum number of solver evaluations of 451. The optimization used the following goals: a reflection coefficient of Γ ≤ −10 dB (40% of weight) at a 3.5 GHz frequency (60% of weight).

**Figure 2:**
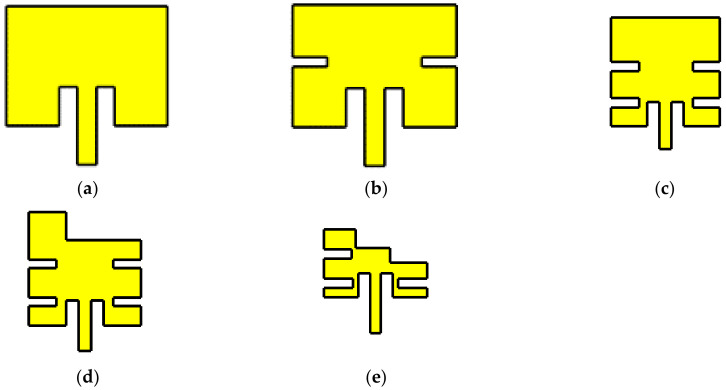
Evolution process of the slot miniaturization technique: (**a**) initial element with 0 slots on the patch, (**b**) 2 slots on the patch, (**c**) 4 slots on the patch, (**d**) 5 slots on the patch, and (**e**) final miniaturized antenna element.

**Figure 3:**
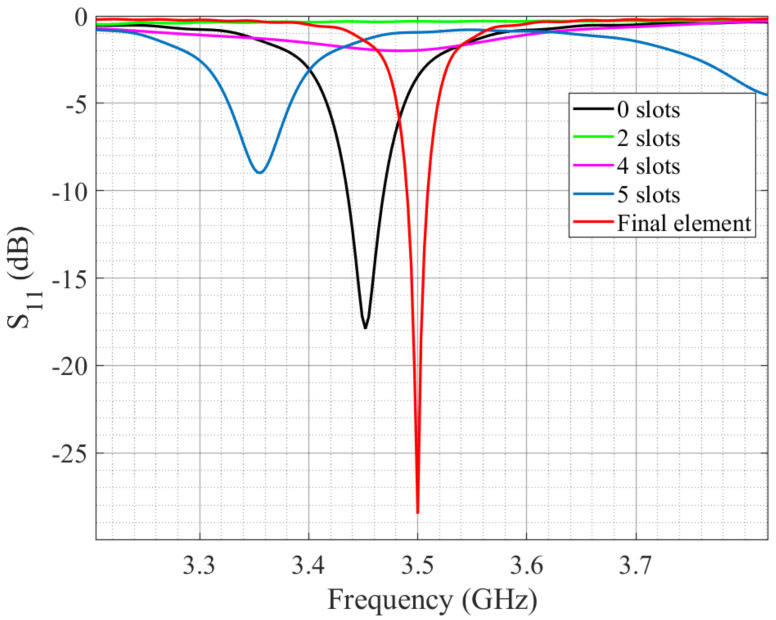
Slot miniaturization technique performance.

Figure 2 shows the evolution process of the miniaturization technique with slots in the antenna element. It is possible to observe the modification of the size and shape of the element. Table 1 presents the gain of each case with the operating wavelength. Meanwhile, Figure 3 exhibits its operation as slots were added to the patch in terms of its reflection coefficient. The slots allowed the antenna element to be reduced in size and set to operate at the desired center frequency of 3.5 GHz rather than the originally designed antenna. The correct operation of the antenna can be observed below −10 dB of the reflection coefficient as the number of slots is increased and the size is parameterized.

**Figure 4:**
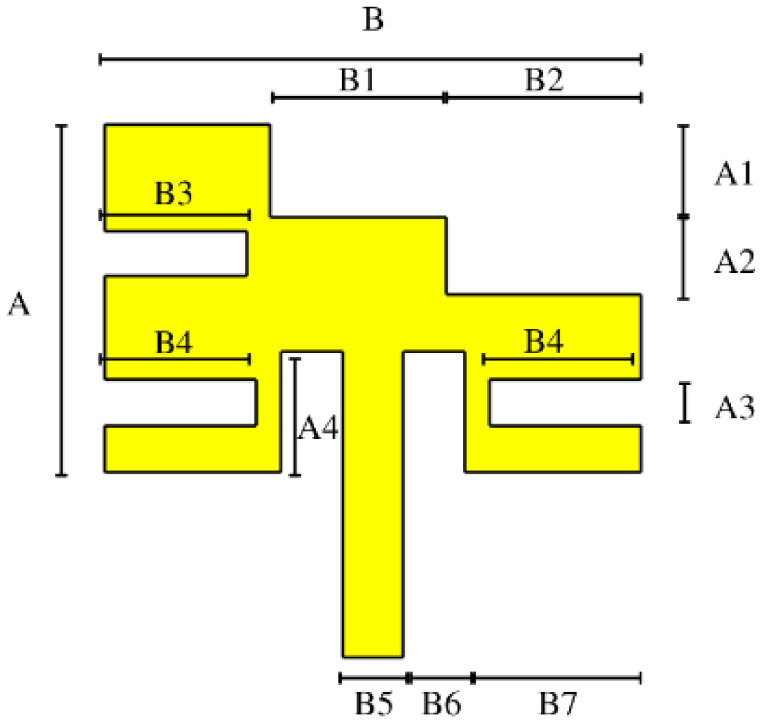
Miniaturized antenna element.

Figure 4 depicts the final optimized element, which resonates at 3.5 GHz. This element uses a Rogers TMM10 substrate with a permittivity of 9.2 and a thickness of 1.270 mm. This substrate was chosen due to its characteristics that allow for great miniaturization. The final dimensions of the proposed miniaturized antenna element are listed in Table 2.

### 2.3. Antenna Array Design

**Figure 5:**
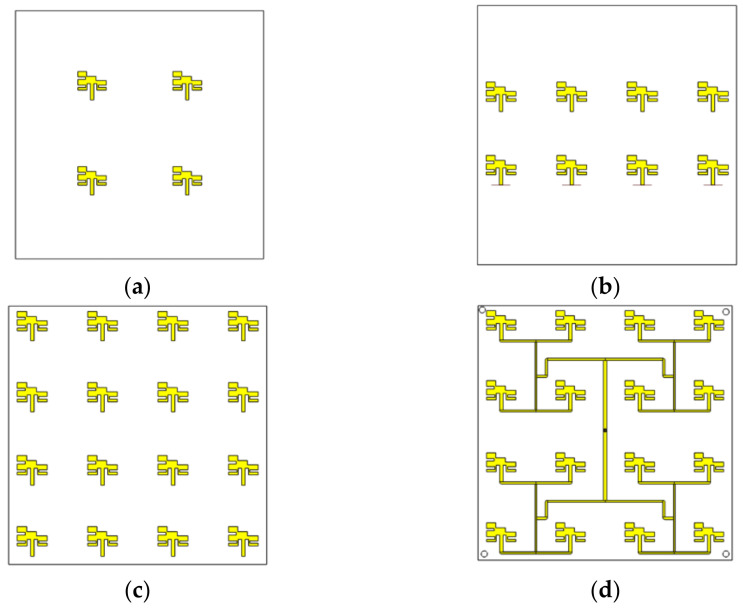
Antenna array evolution: (**a**) 4-element array, (**b**) 8-element array, (**c**) 16-element array, and (**d**) 16-element array with FN.

**Figure 6:**
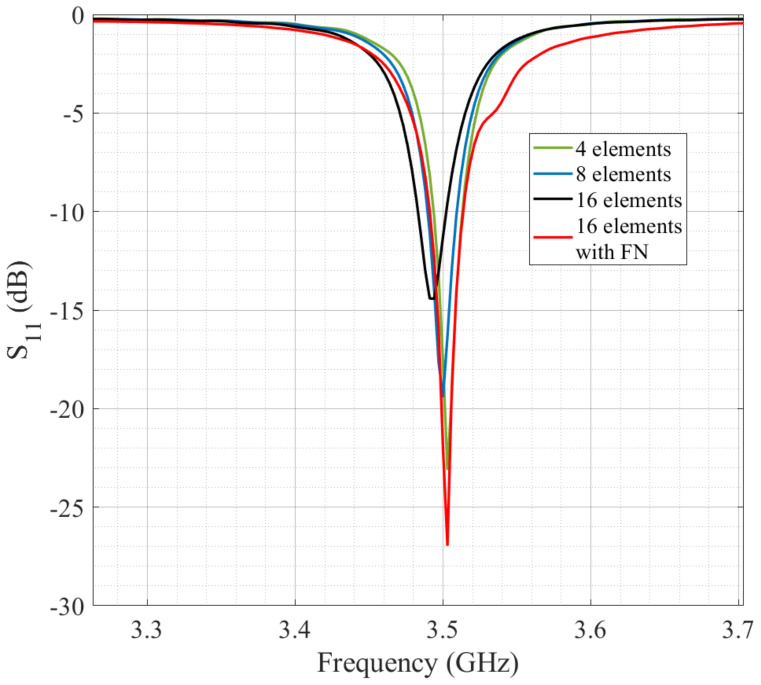
Reflection coefficient comparison of the antenna array evolution.

After the miniaturization of the antenna element, the number of possible elements (N) to form the high-gain antenna array was evaluated by considering the limitations of a 10 × 10 cm substrate for mounting on a CubeSat. Figure 5 shows some of the evaluated topologies. The array cases use 4, 8, and 16 antennas. More than 16 would be impractical due to the reduced space. Mutual coupling affects the array performance with more than 16 elements. Figure 6 illustrates the behavior of the reflection coefficients versus frequency for the analyzed design cases of the array. Table 3 presents the results of each case in terms of gain. It is demonstrated that the best case is 16 antennas since it considers the feeding network (FN) and obtains a high gain.

**Figure 7:**
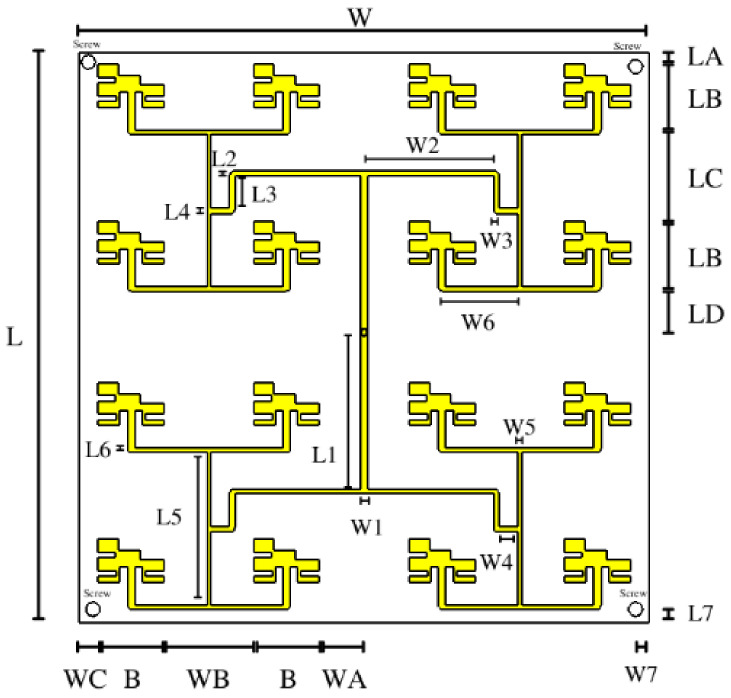
Final antenna array design.

Figure 7 illustrates the final design with the definition of variables for each physical dimension. The elements consider uniform amplitudes and phases with an FN. The CubeSat size restrictions are considered as a base to the size of board L and W, and the constrained spacings are defined as LA ≤ 0.5 λ, LC ≤ 0.5 λ, WB ≤ 0.5 λ, and WC ≤ 0.5 λ. The SMA connector is connected in the center of the array from the other side of the substrate for its feed.

The dimensions of the FN are based on the parameters of the board size and the width of the antenna element [33]. Each dimension of the FN was optimized using CST Studio Suite^®^ software. The details of the optimization strategy were as follows: the particle swarm optimization algorithm was used with a swarm size of 30, a maximum number of iterations of 15, and a maximum number of solver evaluations of 451. The optimization used the following goals: a reflection coefficient of Γ ≤ −10 dB (0.4 of weight) at the 3.5 GHz frequency (0.6 of weight). The final design has the same length from the SMA connector to each antenna element. Table 4 lists the numerical values of each parameter for the array model.

## 3. Research Results

**Figure 8:**
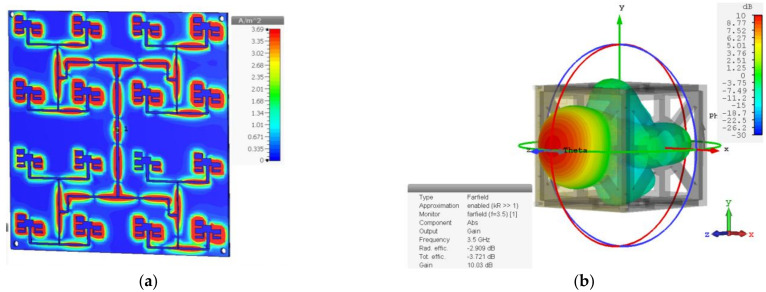
Antenna array design simulation in CST Studio Suite^®^: (**a**) current distribution at 3.5 GHz; (**b**) 3D radiation pattern simulation.

The resulting design is a microstrip patch antenna array with N = 16 elements distributed in a 4 × 4 topology. The current distribution on the antenna array surface in the E-field is presented in Figure 8a. The current flows completely through the FN and reaches each corner of the antennas. Figure 8b depicts a 3D radiation pattern simulation in CST Studio Suite^®^ of the array assembled on a CubeSat structure made of plastic. It can be seen that the radiation pattern is in the broadside direction, which ensures that the radiation will not be wasted in unwanted directions and concentrates it in the center of the array.

**Figure 9:**
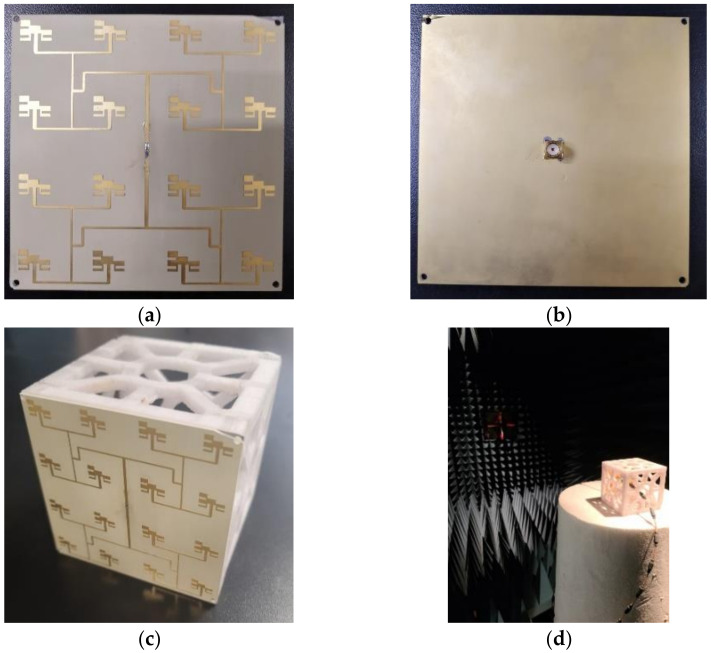
Antenna array prototype: (**a**) top view, (**b**) bottom view, (**c**) prototype assembled on CubeSat, and (**d**) anechoic chamber measurement environment.

Finally, the antenna array was manufactured. Figure 9 shows the prototype that was fabricated and tested in the laboratory. The proposed antenna array was tested using a Keysight PNA-L N5230A vector network analyzer and an ETS Lindgren anechoic chamber, as shown in Figure 9d.

**Figure 10:**
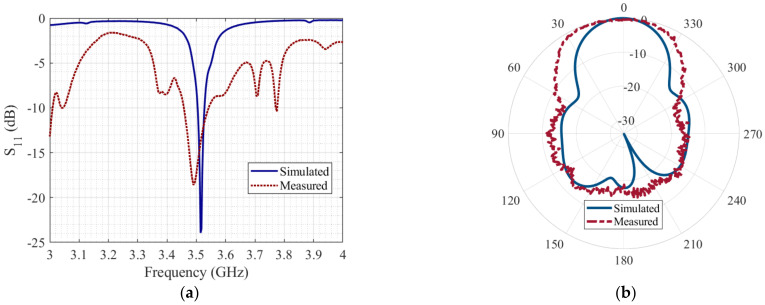
Antenna array performance: (**a**) S_11_ parameter; (**b**) XZ-plane radiation pattern.

Subsequently, a comparative analysis of its performance was carried out. Figure 10 presents the performance of the array in terms of the reflection coefficient and the radiation pattern at 3.5 GHz. The reflection coefficient of the simulated array is under −10 dB from 3.505 to 3.528 GHz. Otherwise, the measured reflection coefficient is under −10 dB from 3.46 GHz to 3.54 GHz, as illustrated in Figure 10a. The purpose of our application case is to operate within the 5G low-frequency band for CubeSat from 3.3 to 4.2 GHz. The welding paste and a solid hold between the SMA connector and the feed line were not included in the simulation. This causes a variation between the simulated and measured reflection coefficient due to technical manufacturing details, which is attributed mainly to fabrication tolerances and discontinuities between the feed line and the SMA junction. Nevertheless, it should be noted that the remarkable relevance of this design is its operation in the established 5G frequency in a low profile. Additionally, Figure 10b shows a comparison of the normalized radiation patterns in the main cut of φ = 0°. In this comparison, we include the radiation pattern of the array simulated in CST Studio Suite^®^ and the measured pattern. The simulated gain of the array mounted on the CubeSat structure was 10.03 dBi, and the measured gain was 8.03 dBi.

Finally, Table 5 lists a comparison of the proposed design with the elements and arrays reported in the literature for CubeSats or the low-frequency band for 5G applications.

Firstly, the low 5G band is used for antennas and arrays in other applications, and they radiate with low gain as reported in [34,35,36,37]. The arrays in [34,36] are focused on multiple-input and multiple-output (MIMO) communications. Moreover, the work presented in [38] has an aperture size slightly larger than 10 cm. These examples cannot be reused in modern nanosatellites.

On the other hand, there exist arrays for CubeSats with higher gains concerning the proposed design in this research. Nevertheless, these arrays are configured for higher frequencies, as shown in [30,39,40]. Some arrays even use more than two ports for MIMO systems in [39,41]. The interesting array in [42] obtains high gain with 256 antennas on a deployable technology that is larger than 10 cm. Finally, the arrays in [43,44] exhibit lower gains in the 2.4 GHz frequency band for CubeSats. Here, the proposed design stands out mainly for considering a low-profile array with high gain performance. This is achieved by using 16 miniaturized elements and an FN in a 5G frequency band. Furthermore, the array was mounted on a CubeSat structure to test its performance. This design can be used for modern CubeSats in 5G applications.

## 4. Conclusions

This paper presents a low-profile antenna array that considers 16 miniaturized patch antennas for CubeSat applications. The innovation of this research lies in the high gain of a microstrip antenna array with FN and its assembly on a CubeSat structure. The main design challenge was the development of an antenna array to operate at low 5G frequencies whilst keeping the overall antenna size smaller than 10 cm × 10 cm. The array was simulated in CST Studio Suite^®^ software and fabricated for performance testing. Due to its reduced dimensions, this design is an excellent alternative for mounting on CubeSat since it combines efficient performance and a low profile. Future work will be focused on the optimization of antenna excitations and the use of aperture couple antennas to increase the gain even more.

## Figures and Tables

**Table 1:** Gain values obtained via the electromagnetic simulation of each analyzed design case of slots in the antenna element.

No. Slots	0	2	4	5	Final Element
Gain (dBi)	5.344	−7.706	2.595	1.993	1.493
Size (*λ*)	0.221	0.221	0.14	0.14	0.1352

**Table 2:** Dimensions of the proposed miniaturized antenna element.

Parameters	A	A1	A2	A3	A4
Values (mm)	7.5	2	1.67	1	2.6
Values (*λ*)	0.0875	0.0233	0.0194	0.0116	0.0303
**Parameters**	**B**	**B1**	**B2**	**B3**	**B4**
Values (mm)	11.592	3.8	4.2	3.0927	3.27
Values (*λ*)	0.1352	0.0443	0.049	0.0360	0.0381
**Parameters**	**B5**	**B6**	**B7**		
Values (mm)	1.32	1.32	3.8164		
Values (*λ*)	0.0154	0.0154	0.0445		

**Table 3:** Gain values obtained via the electromagnetic simulation of each analyzed design case of the array.

No. Elements (N)	4	8	16	16
Feeding Network	Not included	Not included	Not included	Included
Gain (dBi)	6.258	8.012	10.43	10.62

**Table 4:** Array dimensions.

Parameters	L	W	L1	L2	L3	L4	L5	L6
Values (mm)	100	100	27.5	0.8	6.0174	0.995	26.7	0.8
Values (λ)	1.1666	1.1666	0.3208	0.0093	0.0702	0.0116	0.3115	0.0093
**Parameters**	**L7**	**LA**	**LB**	**LC**	**LD**	**W1**	**W2**	**W3**
Values (mm)	2.4	2.003	11.5	16	8	1.255	22.6917	0.935
Values (λ)	0.028	0.0233	0.1341	0.1866	0.0933	0.0146	0.2647	0.0109
**Parameters**	**W4**	**W5**	**W6**	**W7**	**WA**	**WB**	**WC**	
Values (mm)	3.315	0.65	12.6483	2.4	7.8371	15.674	3.3038	
Values (λ)	0.0386	0.0075	0.1475	0.028	0.0914	0.1828	0.0385	

**Table 5:** Comparison of elements and antenna arrays in the literature.

Ref.	Design	Frequency	Bandwidth	Size	Material	Gain	Ports	Applications	CubeSat Structure
[30]	Phased array antenna. N = 16	8.047–8.737 GHz	690 MHz	100 mm × 100 mm 2.736 λ × 2.736 λ	Rogers RT Duroid 5880 and RO3010	High gain Not provided	1	CubeSat	Not included
[34]	Printed monopole antenna. MIMO antenna array. N = 4.	3.37–3.61 GHz	240 MHz	60 mm × 60 mm 0.7 λ × 0.7 λ	Rogers 5870	Between 2.71 dBi and 2.83 dBi	4	WiMax	Not applied
[35]	Microstrip patch antenna. Element.	3.54–3.65 GHz	110 MHz	30 mm × 30 mm 0.36 λ × 0.36 λ	FR4	3.85 dB	1	Wireless	Not applied
[36]	Circular microstrip patch antenna. Element.	3.46–3.57 GHz	110 MHz	30 mm 0.35 λ	FR4	Low gain Not provided	2	Mobile communication	Not applied
[37]	Microstrip patch antenna. Array N = 4.	3.4–3.6 GHz	200 MHz	88.5 mm × 88.5 mm 1.003 λ × 1.003 λ	FR4	5.37 dBi	1	WiFi	Not applied
[38]	Microstrip patch antenna. Array N = 4.	3.7693–3.8413 GHz	72 MHz	Size >100 mm Size >1.26 λ	Rogers Duroid RT5880	13.2 dBi	1	WiMax and UAV	Not applied
[39]	Aperture Coupled Patch Antenna. Array N = 4.	5.48–5.6 GHz	120 MHz	230 mm × 105 mm 4.21 λ × 0.84 λ	Astra MT77	12.4 dBi	2	CubeSat	Not included
[40]	Microstrip patch antenna. Array N = 8.	11.13–12.78 GHz	1.65 GHz	30 mm × 60 mm 2.336 λ × 1.168 λ	FR4	9.37 dB	1	Satellite	Not included
[41]	Slot patch antenna. N = 2.	430–514 MHz	84 MHz	100 mm × 100 mm 0.15 λ × 0.15 λ	Rogers RO4350	Low gain Not provided	2	CubeSat	Not included
[42]	Microstrip patch antenna. Deployable Array N = 256	3.3–3.9 GHz	600 MHz	Size >100 mm Size >1.15 λ	Not provided	30.5 dBi	1	CubeSat	Not included
[43]	Circular microstrip patch antenna. Array N = 4.	2.3–2.5 GHz	200 MHz	100 mm × 100 mm 0.8 λ × 0.8 λ	FR4	4.7 dBi	1	CubeSat	Not included
[44]	Fractal microstrip patch antenna. Element.	2.3–2.5 GHz	200 MHz	72 mm × 72 mm 0.588 λ × 0.588 λ	FR4	3.5 dBi	1	CubeSat	Not included
This work	Microstrip patch antenna. Array N = 16 assembled on CubeSat.	3.46–3.54 GHz	80 MHz	100 mm × 100 mm 1.1666 λ × 1.1666 λ	Rogers TMM10	8.03 dBi	1	CubeSat	Included

## Data Availability

Not applicable.

## References

[1] Fidler F., Knapek M., Horwath J., Leeb W.R. (2010). Optical communications for high-altitude platforms. IEEE J. Sel. Top. Quantum Electron..

[2] Mirza J., Atieh A., Menhas M.I., Ghafoor S., Magam M., Jamal L., Mitu Sheikh S.I., Qureshi K.K. Design of an efficient thulium-doped fiber amplifier for dual-hop earth to satellite optical wireless links. Ain Shams Eng. J..

[3] Rycroff M., Crosby N. (2002). Smaller Satellites: Bigger Business? Concepts, Applications, and Markets for Micro/Nanosatellites in a New Information World.

[4] Hank Heidt H., Puig-Suari J., Moore A.S., Nakasuka S. CubeSat: A new generation of picosatellite for education and industry low-cost space experimentation. Proceedings of the 14th Annual/USU Conference on Small Satellites.

[5] California Polytechnic State University CubeSat Design Specification. https://www.cubesat.org/cubesatinfo.

[6] Tang H., Yang N., Zhang Z., Du Z., Shen J. (2021). 5G NR and Enhancements.

[7] Vaezi M., Azari A., Khosravirad S.R., Shirvanimoghaddam M., Azari M.M., Chasaki D., Popovski P. (2022). Cellular, wide—Area, and Non-Terrestrial IoT: A survey on 5G advances and the road toward 6G. IEEE Comms. Surv. Tutor..

[8] Abulgasem S., Tubbal F., Raad R., Theoharis P.I., Lu S., Iranmanesh S. (2021). Antenna designs for CubeSats: A review. IEEE Access.

[9] Mizuno T.J., Roque J.D., Murakami B.T., Yoneshige L.K., Shiroma G.S., Miyamoto R.Y., Shiroma W.A. Antennas for distributed nanosatellite networks. Proceedings of the IEEE/ACES International Conference on Wireless Communications and Applied Computational Electromagnetics.

[10] Leao T.F.C., Mooney-Chopin V., Trueman C.W., Gleason S. (2013). Design and implementation of a diplexer and a dual-band VHF/UHF antenna for nanosatellites. IEEE Antennas Wireless Propag. Lett..

[11] Prodoningrum R.T., Wijanto H., Prasetyo A.D. Antenna deployment for automatic packet reporting system of nanosatellite using global positioning system as a height sensor. Proceedings of the International Conference on Quality in Research (QiR).

[12] Bellion A., Elis K., De Gaetano S. New compact S-band antenna for nanosatellite telemetry and telecommand applications—EyeSat program. Proceedings of the 10th European Conference on Antennas and Propagation (EuCAP).

[13] Huang T., Reveles J.R., Gurusamy V., Harrington Q., Fraux V. An innovative deployable VHF/UHF helical antenna for nanosatellites. Proceedings of the 13th European Conference on Antennas and Propagation (EuCAP).

[14] El Hammoumi M., Tubbal F., El Amrani El Idrissi N., Raad R., Ioannis Theoharis P., Lalbakhsh A., Abulgasem S. (2022). A wideband 5G CubeSat patch antenna. IEEE J. Miniat. Air Space Syst..

[15] El Hammoumi M., El Amrani El Idrissi N., Raad R., Ioannis Theoharis P., Tubbal F., Abulgasem S. Ultra Wideband dual circularly polarized patch antenna for 5G and CubeSat applications. Proceedings of the 9th International Conference on Wireless Networks and Mobile Communications (WINCOM).

[16] El Hammoumi M., El Amrani El Idrissi N., Raad R., Ioannis Theoharis P., Tubbal F. A wideband compact patch antenna for Ka-band and CubeSat applications. Proceedings of the 15th International Conference on Signal Processing and Communication Systems (ICSPCS).

[17] Aswoyo B., Putra A.H. High gain microstrip square patch array antenna 4 × 4 element 2.3 GHz for 5G communication in Indonesia. Proceedings of the International Electronics Symposium (IES).

[18] Chand Ravi K., Kumar J., Elwi T.A., Mahdi Ali M. Compact MIMO antenna for 5G Applications. Proceedings of the IEEE ANDESCON.

[19] Gupta V., Prabhakar S. Dual band micro-strip patch antennas for 5G sub 6 GHz smart mobile phone and C-band application. Proceedings of the 2nd International Conference on Smart Electronics and Communication (ICOSEC).

[20] Chen X., Wang J., Chang L. (2022). Extremely low profile dual band microstrip patch antenna using electric coupling for 5G mobile terminal application. IEEE Trans. Antennas Propag..

[21] Saxena N. An air substrate microstrip patch antenna for N77 band application. Proceedings of the 2nd Asian Conference on Innovation in Technology (ASIANCON).

[22] Gao Y., Wang J., Wang X., Wei M. (2022). A low profile broadband multimode patch antenna for 5G mobile applications. IEEE Antennas Wirel. Propag. Lett..

[23] Hasan M.M., Rahman R., Shaikh R., Alam I., Islam M.A., Alam M.S. Design and analysis of elliptical microstrip patch antenna at 3.5 GHz for 5G applications. Proceedings of the IEEE Region 10 Symposium (TENSYMP).

[24] Magalhães M.P., Heckler M.V.T., Mota J.C.M., Sombra A.S.B., Moreira E.C. Design and analysis of microstrip antenna arrays for meteorological nano-satellites for UHF uplink. Proceedings of the International Telecommunications Symposium (ITS).

[25] Vieira J.M., Yoshimoto E., Ferreira F.G., Pereira V.M., Heckler M.V.T. UHF and S-band antenna arrays for nano-satellite-based data-relay. Proceedings of the 12th European Conference on Antennas and Propagation (EuCAP 2018).

[26] Alam T., Islam M.T., Ullah M.A., Rahmatillah R., Aheieva K., Lap C.C., Cho M. (2018). Design and compatibility analysis of a solar panel integrated UHF antenna for nanosatellite space mission. PLoS ONE.

[27] Benyamin S.O., Wijanto H., Prabowo V.S.W., Prananditya H., Oktaviani S.M. Design and Characterization Of Rectangular Array Microstrip Antenna For Cubesat S-Band Transmitter. Proceedings of the 3rd International Conference on Information and Communications Technology (ICOIACT).

[28] Arnaud E., Menudier C., Fouany J., Monediere T., Thevenot M. (2017). X-band compact dual circularly polarized isoflux antenna for nanosatellite applications. Int. J. Microw. Wirel. Technol..

[29] Arnaud E., Siblini A., Bellion A., Jecko B. (2020). Experimental validation of an isoflux Earth coverage with a bimode ARMA antenna on a nanosatellite. Int. J. Microw. Wirel. Technol..

[30] Hashim I.S.M., Al-Hourani A., Wayne Rowe S.T., Scott J.R. Adaptive X-band satellite antenna for Internet-of-Things (IoT) over satellite applications. Proceedings of the 13th International Conference on Signal Processing and Communication Systems (ICSPCS).

[31] Squadrito P., Zhang S., Pedersen J.F. High gain K-band patch antenna for low earth orbit interlink between nanosatellites. Proceedings of the 12th European Conference on Antennas and Propagation (EuCAP 2018).

[32] Balanis C.A. (2005). Microstrip Antennas. Antenna Theory. Analysis and Design.

[33] Stutzman W.L., Thiele G.A. (2012). Antenna Theory and Design.

[34] Mishra M., Chaudhuri S., Kshetrimayum R.S. Low mutual coupling four-port MIMO antenna array for 3.5 GHz WiMAX application. Proceedings of the IEEE Region 10 Symposium (TENSYMP).

[35] Rajeshkumar V., Raghavan S. A compact CSRR loaded dual band microstrip patch antenna for wireless applications. Proceedings of the IEEE International Conference on Computational Intelligence and Computing Research.

[36] Wang Y., Piao D. A dual-polarized antenna with pattern diversity based on a two-mode single-layer microstrip patch. Proceedings of the IEEE MTT-S International Wireless Symposium (IWS).

[37] Chen W.S., Lin Y.S. Design of 2 × 2 microstrip patch array antenna for 5G C-band access point applications. Proceedings of the IEEE International Workshop on Electromagnetics: Applications and Student Innovation Competition (iWEM).

[38] Sajjad H., Sethi W.T., Zeb K., Mairaj A. Microstrip patch antenna array at 3.8 GHz for WiMax and UAV applications. Proceedings of the International Workshop on Antenna Technology: Small Antennas, Novel EM Structures and Materials, and Applications (iWAT).

[39] Bouça P., Matos J.N., Cunha S.R., Carvalho N.B. (2020). Low-profile aperture-coupled patch antenna array for CubeSat applications. IEEE Access.

[40] Harane M.M., Ammor H. Design & development of 4 × 2 microstrip patch antenna array with circular polarized elements for satellite application. Proceedings of the International Symposium on Advanced Electrical and Communication Technologies (ISAECT).

[41] Hussain R., Rao A.S., Aziz A., Khan M.U., Sharawi M.S. Highly miniaturized folded-slot based MIMO antenna design for CubeSat applications. Proceedings of the 16th European Conference on Antennas and Propagation (EuCAP).

[42] Warren P.A., Steinbeck J.W., Minelli R.J., Mueller C. Large, deployable S-band antenna for a 6U CubeSat. Proceedings of the 29th Annual AIAA/USU Conference on Small Satellites.

[43] Figueroa Torres C.A., Medina Monroy J.L., Lobato Morales H., Chávez Pérez R.A., Calvillo Téllez A. (2016). Microstrip circular antenna array design for CubeSat applications. Aristas J. Basic Appl. Sci..

[44] Figueroa Torres C.A., Medina Monroy J.L., Lobato Morales H., Chávez Pérez R.A., Calvillo Téllez A. (2016). A microstrip antenna based on a standing-wave fractal geometry for CubeSat applications. Microw. Opt. Technol. Lett..

